# Circulating galectin-3 level association with cardiovascular risk factors during peritoneal dialysis

**DOI:** 10.1007/s10157-024-02498-3

**Published:** 2024-04-20

**Authors:** Xuerui Yang, Jun Yang, Youjia Zeng, Ling Peng, Xingzheng Liu, Jinying Mo, Taifen Wang, Yutong Yao, Yihou Zheng, Gaofeng Song

**Affiliations:** Department of Nephrology, Shenzhen Traditional Chinese Medicine Hospital, The Fourth Clinical Medical College of Guangzhou University of Chinese Medicine, 1 Fuhua Road, Futian District, Shenzhen, 518033 Guangdong China

**Keywords:** Chronic kidney disease, Peritoneal dialysis, Cardiovascular disease, Galectin-3

## Abstract

**Objective:**

Cardiovascular disease (CVD) represents the primary cause of mortality in patients afflicted with end-stage renal disease and undergoing peritoneal dialysis (PD) treatment. Galectin-3 (Gal-3), a molecule known to exhibit a correlation with CVD mortality garners considerable interest. The objective of this study was to explore the potential association between serum Gal-3 levels and other CVD risk factors among PD patients.

**Methods:**

In this cross-sectional study, a total of 114 PD patients with a minimum of 3 months of PD treatment were enrolled. Serum Gal-3 levels were quantified using an enzyme-linked immunosorbent assay. The data of patients with Gal-3 levels higher and lower than 26.744 pg/ml were compared using Mann–Whitney *U* tests or *t* tests. Pearson’s correlation or Spearman’s correlation analysis and multivariate regression were used to assess the associations between the known risk factors for CVD and Gal-3.

**Results:**

In comparison to the inter-group baseline data, the low Gal-3 group exhibited a higher glomerular filtration rate (GFR). Gal-3 levels correlate positively with PD duration, B-type natriuretic peptide (BNP), growth differentiation factor 15 (GDF-15), interventricular septal thickness in diastolic (IVST), and left ventricular mass index (LVMI). Conversely, Gal-3 exhibited a negative correlation with albumin levels. Multivariate linear regression analysis demonstrated a positive correlation between Gal-3 levels and BNP, GDF-15, PD duration, IVST and LVMI. Gal-3 levels were negatively correlated with albumin levels.

**Conclusions:**

Gal-3 was strongly associated with BNP, GDF-15, IVST and LVMI in patients undergoing PD treatment. Prospective studies should be carried out to determine whether Gal-3 can be a promising biomarker in predicting increased risk of adverse cardiovascular events in PD patients.

## Introduction

Chronic kidney disease (CKD) represents a substantial clinical and public health challenge due to its escalating incidence and prevalence rates. It stands as a prominent contributor to both morbidity and mortality associated with end-stage renal disease (ESRD) on a global scale [1]. Peritoneal dialysis (PD) serves as a prevalent home-based therapeutic approach for patients with ESRD, accounting for 11% of all dialysis cases and 9% of all kidney replacement therapy worldwide [2]. Among patients undergoing PD for ESRD, cardiovascular disease (CVD) stands out as a primary cause of mortality. Notably, the mortality rate due to CVD in individuals undergoing dialysis is 10 to 20 times higher compared to the general population [3]. In light of these circumstances, early identification and management of the high-risk subgroup within the PD patient population, based on the stratification of early CVD risk and understanding the underlying mechanisms, become paramount. However, traditional risk factors do not fully explain the heightened mortality observed in PD patients [4]. Therefore, the identification of potential diagnostic targets holds paramount importance in the pursuit of improving CVD morbidity and mortality among patients undergoing PD.

Galectin-3 (Gal-3) is a 31 kDa member of a growing family of β-galactoside-binding animal lectins [5]. It serves as a maker of fibrosis and inflammation, contributing to cardiac fibroblast proliferation, collagen deposition, and ventricular dysfunction [6]. Notably, Gal-3 has emerged as a significant independent predictor for all-cause mortality, adverse cardiovascular events, and left ventricular remodeling in the general population [7]. The Food and Drug Administration has approved the use of Gal-3 assay together with clinical assessment for assessing the prognosis of chronic heart failure (HF) [8]. Gal-3 also has been validated as a novel biomarker for predicting CVD mortality in patients on maintenance hemodialysis (HD) [9]. However, its potential role in predicting CVD risk in PD patients remains undefined.

Thus, this study determined the Gal-3 levels among patients with maintenance PD. We aimed to investigate the association between Gal-3 levels and known risk factors for CVD. The outcomes of our research endeavor will offer valuable insights for enhancing the management strategies employed in PD patients.

## Materials and methods

### Subjects

Our study took place between May 2022 and May 2023 at the outpatient PD unit at Shenzhen Traditional Chinese Medicine Hospital, in Guangdong, China.

**Fig. 1:**
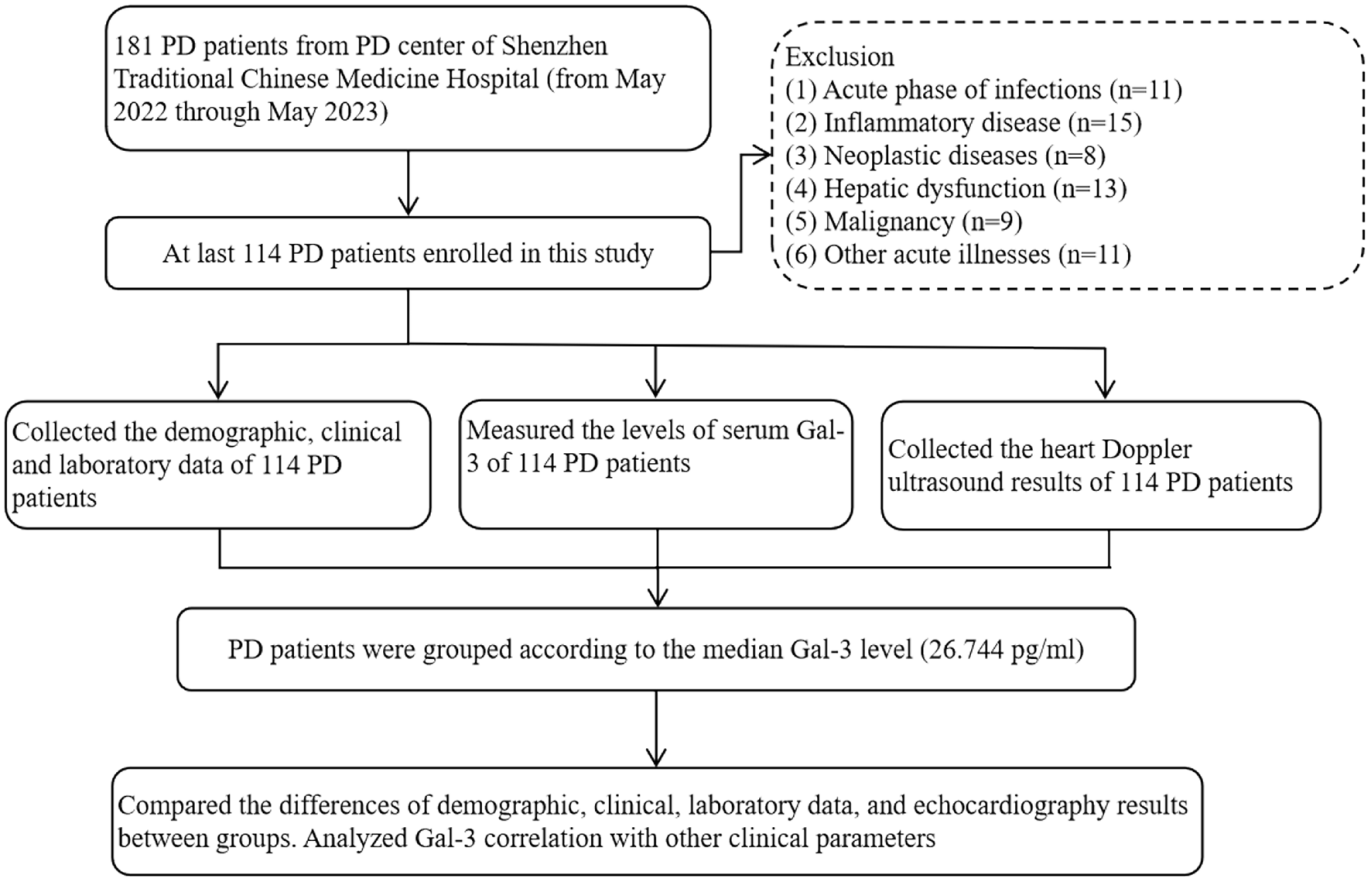
Flowchart of the study process. *PD* peritoneal dialysis, *Gal-3* galectin-3

This cross-sectional study enrolled a total of 114 patients undergoing PD (Fig. 1). The inclusion criteria encompassed adults above 18 years of age who had been receiving continuous ambulatory PD for a minimum of 3 months, with 3–5 manual exchanges daily using 2000 ml solutions. Patients with acute infections, inflammatory diseases, neoplastic diseases, hepatic dysfunction, malignancy, or other acute illnesses were excluded. The Shenzhen Traditional Chinese Medicine Hospital’s Medical Ethics Committee provided authorization for this project (Grant number: K2022-057-01). All participants were informed about the study protocol, and written informed consent was obtained prior to their involvement.

The patients who met the inclusion criteria were examined clinically and with echocardiography. All participants underwent venous blood sampling immediately prior to the echo study. Blood samples were spun and frozen at − 80 °C and analyzed subsequently using standardized commercially available kits.

### Clinical data collection

Demographic data of patients, such as age, gender, height, weight, body mass index (BMI), PD duration, diabetes mellitus, systolic blood pressure (SBP), diastolic blood pressure (DBP), primary kidney disease, comorbidities, and medication, were extracted from medical records.

Blood samples were taken from a peripheral vein in the morning, following a fasting period of over 8 h. Biochemical measurements were assessed using conventional laboratorial techniques for clinical use. What’s more, the fractional clearance index for urea (Kt/V), glomerular filtration rate (GFR), and residual renal function (RRF) were also included in the statistical analysis. GFR was estimated by using the simplified modification of diet in renal disease study (MDRD) equation. eGFR = 186.3 × serum creatinine^−1.154^ × age^−0.203^ × (0.742 for women) ml·min^−1^·(1.73 m^2^)^−1^. RRF was estimated by calculating the mean of the 24 h creatinine clearance and urea clearance.

### Determination of serum biomarkers

The blood samples collected in a fasting state were promptly frozen and stored at a temperature of − 80 °C until analysis. Serum levels of Gal-3 and growth differentiation factor 15 (GDF-15) were measured using an enzyme-linked immunosorbent assay (ELISA), following the guidelines provided by the manufacturer (R&D Systems, Minneapolis, MN, USA).

### Transthoracic echocardiography

Echocardiography measurements were conducted by an experienced cardiologist who was unaware of the patients’ clinical data. Standard apical four-chamber, parasternal long and short axis views of the left ventricular were utilized to measure echocardiographic parameters. The following parameters were measured using two-dimensional guided M-mode and Doppler echocardiography, in accordance with the recommendations for chamber quantification: left atrial end-systolic diameter (LADs), left ventricular end-diastolic diameter (LVDd), interventricular septal thickness in diastole (IVST), and posterior wall thickness in diastole (PWT). In addition, left ventricle ejection fraction (LVEF), left ventricular fractional shortening (LVFS), and left ventricular mass index (LVMI) were evaluated in all participants.

### Statistical methods

Statistical analysis was performed using Statistical Product and Service Solutions (SPSS, Version X; IBM, Armonk, NY, USA) 20.0 software. Non-normally distributed variables were presented as median with interquartile range (IQR), while normally distributed variables were expressed as mean ± standard deviation (SD). The patients were divided into two groups based on the median value of plasma Gal-3. The two groups were compared using appropriate statistical tests such as the *t* test or Mann–Whitney *U* test. Pearson’s correlation coefficient was used for continuous variables with normal distribution and Spearman’s correlation coefficient was used for continuous variables that were not normally distributed. Furthermore, we conducted multivariable analyses with adjustment for age, gender, diabetes mellitus and history of cardiovascular event to evaluate the association between Gal-3 levels in PD patients and known risk factors for CVD. A significance level of* P* < 0.05 was considered statistically significant.

## Results

### Baseline characteristics of patients according to Gal-3 median

The demographic and laboratory data of the 114 PD patients in the study are presented in Table 1. The average age of the patients was 46.46 years, and 64.9% of them were male. The median duration of PD therapy was 12.0 (7.0–24.0) months. Among the participants, 24 individuals (21.0%) had diabetes. The median Kt/V value was 2.08 (1.77–2.4). In terms of therapeutics, 9.6% of the patients received angiotensin-converting enzyme inhibitor (ACEI), 70.1% were treated with angiotensin II receptor blocker (ARB), 82.4% received calcium channel blocker (CCB), 80.7% were prescribed β-blocker, and 18.4% were on statin.

**Table 1:** Demographic, clinical characteristics and laboratory findings of PD patients stratified by the median plasma levels of Gal-3

Variables	All patients (*n* = 114)	Gal-3 < 26.744 (*n* = 57)	Gal-3 ≥ 26.744 (*n* = 57)	*P* value
Age (years)	46.46 ± 11.93	44.86 ± 12.31	48.07 ± 11.42	0.152
Gender (male, *n*, %)	74 (64.9%)	42 (73.7%)	32 (56.1%)	0.088
PD duration (months)	12 (7–24)	12.0 (7.5–15)	14 (4.5–39)	0.102
Diabetes mellitus (%)	24 (21.0%)	11 (19.2%)	13 (22.8%)	0.646
SBP (mmHg)	144.84 ± 21.81	143.67 ± 19.88	146.02 ± 23.69	0.567
DBP (mmHg)	90.78 ± 12.89	90.32 ± 13.50	91.25 ± 12.35	0.702
Kt/V	2.08 (1.77–2.40)	2.07 (1.76–2.36)	2.08 (1.77–2.41)	0.802
RRF(ml/min)	19.39 ± 11.72	20.23 ± 11.08	18.41 ± 12.48	0.271
**GFR (ml/min/1.73 m**^**2**^**)**	**5.27 ± 1.57**	**5.60 ± 1.32**	**4.90 ± 1.70**	**0.015**
ACEI (*n*, %)	11 (9.6%)	5 (8.7%)	6 (10.5%)	0.751
ARB (*n*, %)	80 (70.1%)	42 (73.6%)	38 (66.6%)	0.413
CCB (*n*, %)	94 (82.4%)	45 (78.9%)	49 (85.9%)	0.325
β-Blocker (*n*, %)	92 (80.7%)	44 (77.1%)	48 (84.2%)	0.342
Statin (*n*, %)	21 (18.4%)	7 (12.2%)	14 (24.5%)	0.091
TG (mg/dl)	117.40 (83.95–158.74)	117.84 (83.28–158.50)	111.64 (87.71–149.74)	0.975
TC (mg/dl)	164.54 ± 37.90	165.99 ± 36.82	163.10 ± 39.22	0.685
LDL-C (mg/dl)	98.36 ± 33.91	101.48 ± 32.20	95.24 ± 35.56	0.328
HDL-C (mg/dl)	39.63 (33.55–49.11)	39.82 (33.25–45.63)	39.06(35.19–53.36)	0.282
Scr (mg/dl)	10.24 (8.96–13.10)	9.58 (8.74–12.47)	11.08 (9.20–13.81)	0.055
Hemoglobin (g/dl)	10.40 (8.88–73.00)	9.40 (8.50–10.35)	9.20 (8.30–10.50)	0.998
Albumin (g/dl)	3.32 (1.76–3.68)	3.42 (2.78–3.79)	3.13 (1.70–3.66)	0.087
BNP (pg/ml)	121.00 (49.50–280.00)	130.00 (52.70–242.50)	107.00 (37.90–333.50)	0.832
**GDF-15(ng/ml)**	**3.63 (2.67–5.03)**	**3.21 (2.45–4.34)**	**3.99 (2.96–5.56)**	**0.008**
LADs (mm)	32 (29–36.25)	32 (29–33)	33(30–37)	0.147
LVDd (mm)	46 (42–49)	46 (43–48)	46.50 (42–50)	0.776
IVST (mm)	11 (10–12)	11 (10–12)	11 (10–12.38)	0.121
PWT (mm)	11 (10–12)	11 (10–11)	11 (10–12)	0.360
LVEF (%)	64.5 (62–66)	65 (62–66)	64 (63–66)	0.923
LVFS (%)	34 (32–36)	35 (33–36)	34 (32–36)	0.637
LVMI (g/m^2^)	107.12 (88.49–128.57)	104.89 (88.72–117.63)	115.89 (87.97–142.79)	0.102

The variables in bold are statistically significant

*BMI* body mass index, *PD* peritoneal dialysis, *SBP* systolic blood pressure, *DBP* diastolic blood pressure, *Kt/V* fractional clearance index for urea, *RRF* residual renal function, *GFR* glomerular filtration rate, *ACEI* angiotensin-converting enzyme inhibitor, *ARB* angiotensin II receptor blockers, *CCB* calcium channel blockers, *TG* triglyceride, *TC* total cholesterol, *LDL-C* low-density lipoprotein cholesterol, *HDL-C* high-density lipoprotein cholesterol, *Scr* serum creatinine, *BNP* B-type natriuretic peptide, *GDF-15* growth differentiation factor 15, LADs left atrial end-systolic diameter, *LVDd* left ventricular end-diastolic diameter, *IVST* interventricular septal thickness in diastolic, *PWT* posterior wall thickness in diastolic, *LVEF* left ventricle ejection fraction, *LVFS* left ventricular fractional shortening, *LVMI* left ventricular mass index

To assess the correlation between Gal-3 and clinical and laboratory findings in patients undergoing maintenance PD, we split the patient cohort into 2 subsets (*n* = 57) according to median Gal-3 (26.744 pg/ml). The low Gal-3 group exhibited a higher GFR (*P* = 0.015). Furthermore, patients with higher Gal-3 concentrations showed increased levels of GDF-15 (*P* = 0.008).

### Association between Gal-3 and cardiovascular risk factors

Pearson’s correlation coefficient and Spearman’s rank correlation coefficients were utilized to confirm the correlation between Gal-3 and several cardiovascular risk factors in maintenance PD patients (Table 2). Serum Gal-3 levels were found to have a positive association with PD duration (*r* = 0.283, *P* = 0.002), BNP (r = 0.257, *P* = 0.006), GDF-15 (*r* = 0.272, *P* = 0.003), IVST (*r* = 0.260, *P* = 0.005), LVMI (*r* = 0.208, *P* = 0.027) while exhibiting a negative correlation with albumin levels (*r* = − 0.274, *P* = 0.003).

**Table 2:** Gal-3 correlation with cardiovascular risk factors among PD cases

Variables	correlation coefficient	*P* value
Age (years)	0.141	0.135
**PD duration(months)**	**0.283**	**0.002**
SBP (mmHg)	0.171	0.068
DBP (mmHg)	0.102	0.279
TG (mg/dl)	0.023	0.810
TC (mg/dl)	− 0.011	0.904
LDL-C (mg/dl)	− 0.088	0.349
HDL-C(mg/dl)	0.152	0.106
Hemoglobin (g/dl)	− 0.014	0.886
**Albumin (g/dl)**	− **0.274**	**0.003**
**BNP (pg/ml)**	**0.257**	**0.006**
Kt/V^*^	0.017	0.873
RRF	0.040	0.704
GFR (ml/min/1.73 m^2^)	− 0.172	0.067
**GDF-15(ng/ml)**	**0.272**	**0.003**
LADs (mm)	0.163	0.083
LVDd (mm)	− 0.028	0.765
**IVST (mm)**	**0.260**	**0.005**
PWT (mm)	0.133	0.160
LVEF (%)	− 0.024	0.796
LVFS (%)	− 0.055	0.558
**LVMI (g/m**^**2**^**)**	**0.208**	**0.027**

The variables in bold are statistically significant

### Multivariate linear regression analysis of the association of serum Gal-3 levels with cardiovascular risk factors

Those parameters which are well-known risk factors, such as BNP, Albumin, PD duration, GDF-15, RRF, LADs, LVDd, IVST, PWT, LVEF, LVFS and LVMI, were all included in multivariate linear regression analyses (Table 3). In this study, the association of galectin-3 with other CVD risk factors was adjusted for age, gender, diabetes mellitus and history of cardiovascular event. The results indicated a significant positive correlation between Gal-3 levels and BNP (*β* = 0.267, *P* = 0.006), PD duration (*β* = 0.269, *P* = 0.004), GDF-15 (*β* = 0.276, *P* = 0.005), IVST (*β* = 0.259, *P* = 0.006) and LVMI (*β* = 0.202, *P* = 0.034). Additionally, Gal-3 levels were negatively correlated with albumin levels (*β* = − 0.327, *P* = 0.001).

**Table 3:** Multivariate linear regression analysis of the association of serum Gal-3 levels with cardiovascular risk factors

Parameters^a^	Regression coefficient	95% CI	*P* value
**BNP**	**0.267**	**0.002**–**0.009**	**0.006**
**Albumin**	− **0.327**	− **5.388** to − **1.326**	**0.001**
**PD duration**	**0.269**	**0.039**–**0.209**	**0.004**
**GDF-15**	**0.276**	**0.456**–**2.581**	**0.005**
RRF	− 0.035	− 0.213–0.152	0.739
LADs	0.147	− 0.090–0.677	0.133
LVDd	0.031	− 0.351–0.254	0.750
**IVST**	**0.259**	**0.176**–**1.040**	**0.006**
PWT	0.123	− 0.526–2.385	0.208
LVEF	− 0.041	− 0.534–0.344	0.670
LVFS	− 0.060	− 0.814–0.419	0.528
**LVMI**	**0.202**	− **0.005**–**0.125**	**0.034**

The variables in bold are statistically significant

^a^Adjusted for age, gender and diabetes mellitus and history of cardiovascular event

## Discussion

Our findings present novel insights into the relationships between serum Gal-3 levels and other CVD risk factors in patients undergoing PD. Specifically, we found that Gal-3 was strongly positively associated with BNP, GDF-15, IVST and LVMI in patients treated for PD, suggesting that Gal-3 may be a promising predictor of cardiovascular event risk in patients with PD.

Gal-3 plays a vital role in embryonic development, inflammation, and fibrosis promotion [10]. It interacts with galactose-containing glycoproteins on the cell surface, regulating various activities and signaling pathways related to cell proliferation, migration, adhesion, and cell–cell interactions [11]. Gal-3 was found to be associated with incident CKD, particularly among individuals who had hypertension at baseline [12]. Aldosterone could induce Gal-3 secretion in vitro and in vivo, which has been implicated in the development of fibrosis of several organs, including the kidney, through fibroblast proliferation and extracellular matrix remodeling [13–15]. Meanwhile, Gal-3 is associated with cardiac fibrosis, ventricular remodeling, and dysfunction [16]. The elevated burden of CVD in populations with renal disease is due to its role as both a significant cause and consequence of CKD. A prospective longitudinal observational study has confirmed that Gal-3 levels are significantly elevated in PD patients and are significantly associated with PD failure [17]. This may be due to the gradual fibrosis of the peritoneum and the recruitment of inflammatory cells under the stimulation of non-physiological PD solution for a long time [18]. Peritoneal status is also closely related to the vulnerability of the cardiovascular system and the nutritional status of patients [17, 19, 20]. The results of this study are consistent with previous studies but more prospective controlled studies are needed to elucidate whether Gal-3 may represent a novel cytokine predicts CVD in PD patients.

GDF-15 is a divergent member of the transforming growth factor β superfamily, is a stress-responding cytokine whose expression levels respond to a variety of cellular stress signals, such as inflammation, hypoxia, tissue injuries and myocardial ischemia [21]. In a specific range, circulating GDF-15 independently predicted CKD and CVD progression and worse prognosis [9, 22, 23]. In a study of 625 patients on maintenance HD in Vienna, Austria by Stephan Nopp et al. [24], GDF-15 is a promising biomarker to be included in death and cardiovascular risk assessment in patients with HD. And its close interaction with kidney disease progression, inflammation, ischaemia and endothelial dysfunction may explains its strong predictive capability as a cardiovascular biomarker in end-stage kidney disease (ESKD) patients with HD. In Correlation analysis of this study, Gal-3 and GDF-15 are significantly positively correlated. Gal-3 and GDF-15 share common characteristics, e.g., both are related to fibrosis [6, 21]. As progressive cardiac fibrosis is a pivotal aspect in the development of cardiac dysfunction and a potential trigger for lethal arrhythmias and sudden death, the presence of a blood marker for cardiac fibrosis would undoubtedly serve as a crucial prognostic indicator for survival and cardiovascular events [9]. In multivariate linear regression analysis, the correlation between Gal-3 and GDF-15 was also statistically significant. Therefore, Gal-3 and GDF-15 may be used together to predict the risk of CVD in the PD cohort.

BNP, a peptide hormone, is secreted by ventricular myocytes in response to myocardial stretch induced by elevated cardiac filling pressure [25, 26]. It serves as a recognized biomarker for diagnosing and prognosticating heart failure [27]. Rutten et al. [28] found PD patients with BNP above the median (7.5 pmol/l) had significantly increased mortality compared to patients with BNP under the median. The presence of elevated serum BNPs is prevalent among chronic PD patients, yet it demonstrates a robust and independent correlation with cardiovascular morbidity as well as overall and cardiovascular mortality [29]. It was found in an experimental study of Fermann et al.[30] that Gal-3 in some acute heart failure patients is higher than the level predicted by their own BNP. BNP is a kind of neurohormone secreted by the heart when the heart is overloaded and Gal-3 can promote the myocardial fibrosis and myocardial apoptosis. Therefore, Gal-3 may serve as a connection between inflammation and the neurohormonal system in CVD [31]. In this study, multivariate linear regression analysis confirmed that Gal-3 levels were positively correlated with BNP. The use of Gal-3 and BNP together probably permits a better biomarker for predicting CVD in PD patients. The value of this approach has not been clearly demonstrated in PD patients and will require further study.

In this study, we also examined the associations between Gal-3 and echocardiographic indicators. Multivariate linear regression analysis confirmed that Gal-3 levels were positively correlated with IVST and LVMI. What’s more, previous studies found that galectin-3 is directly related to the cardiac remodeling [32]. Thus, combining Gal-3 with BNP and echocardiography may be a better strategy for predicting the risk of cardiovascular events in PD patients.

Several limitations should be considered. It is a cross-sectional study without a healthy control group. We view it as a preliminary investigation into Gal-3’s role in predicting increased cardiovascular risk in PD patients. And our PD patients were from one Chinese hospital, so generalizing findings to patients in other countries is unclear. Future longitudinal studies with healthy controls and larger sample sizes are needed.

In summary, this preliminary study showed elevated Gal-3 levels in PD patients, which positively correlated with GDF-15, BNP, IVST and LVMI. Well-designed prospective studies are needed to determine whether Gal-3 could potentially serve as a biomarker for early cardiovascular event diagnosis in this patient population and predict increased risk during PD treatment.
